# TPMT、NUDT15基因多态性对成人急性淋巴细胞白血病患者6-巯基嘌呤耐受性的影响

**DOI:** 10.3760/cma.j.issn.0253-2727.2021.11.005

**Published:** 2021-11

**Authors:** 其姗 郝, 哲 王, 秋云 房, 晓媛 弓, 凯奇 刘, 艳 李, 辉 魏, 迎 王, 庆华 李, 敏 王, 征 田, 建祥 王, 营昌 秘

**Affiliations:** 中国医学科学院、北京协和医学院血液病医院（中国医学科学院血液学研究所），实验血液学国家重点实验室，国家血液系统疾病临床医学研究中心，天津 300020 Institute of Hematology & Blood Diseases Hospital, Chinese Academy of Medical Sciences & Peking Union Medical College, State Key Laboratory of Experimental Hematology, National Clinical Research Center for Blood Diseases, Tianjin 300020

**Keywords:** 白血病，淋巴样, 成年人, 6-巯嘌呤, 基因多态性, Leukemia, lymphoid, Adult, 6-Mercaptopurine, Polymorphism, gene

## Abstract

**目的:**

探讨TPMT*2 rs1800462、TPMT*3B rs1800460、TPMT*3C rs1142345和NUDT15 rs116855232基因多态性对成人急性淋巴细胞白血病（ALL）患者6-巯基嘌呤（6-MP）耐受性的影响。

**方法:**

选取2015年9月至2019年12月于中国医学科学院血液病医院确诊并接受环磷酰胺、阿糖胞苷和6-MP（CAM方案）治疗的216例成人ALL患者，利用Taqman SNP基因分型方法检测患者的TPMT、NUDT15基因型。结合临床资料，分析基因多态性对ALL治疗中含6-MP方案耐受性的影响。

**结果:**

216例患者中B-ALL 185例（85.65％）、T-ALL 31例（14.35％）。TPMT*2 rs1800462 CC型216例（100.00％）；TPMT*3B rs1800460 CC型216例（100.00％）；TPMT*3C rs1142345 TT型209例（96.76％）、TC型7例（3.24％），等位基因突变频率为1.62％。NUDT15 rs116855232 CC型166例（76.85％）、CT型48例（22.22％）、TT型2例（0.93％），等位基因突变频率为12.04％。TPMT*3C rs1142345突变型组（TC+CC型）悬浮红细胞的输注量少于野生型组（CC型）（*P*＝0.036）；TPMT*3C rs1142345突变型组出现肝损害（天冬氨酸转氨酶升高）的风险高于野生型组（*OR*＝9.559, 95％*CI* 1.135～80.475, *P*＝0.038）。NUDT15 rs116855232突变型组（CT+TT型）WBC<1×10^9^/L和中性粒细胞绝对计数<0.5×10^9^/L的持续时间均长于野生型组（CC型）（*P*＝0.005，*P*＝0.007）；突变型组单采血小板的输注量多于野生型组（*P*＝0.014）。

**结论:**

TMPT、NUDT15基因多态性可以影响成人ALL患者对6-MP的耐受性。治疗前检测患者基因型，进而优化6-MP使用剂量，有助于缩短骨髓抑制时间和减少血制品输注量。临床试验注册：中国临床试验注册中心（ChiCTR-TNC-09000397）

## Abstract (en)

**Objective:**

To investigate the effect of genetic polymorphisms of TPMT*2 rs1800462, TPMT*3B rs1800460, TPMT*3C rs1142345, and NUDT15 rs116855232 on the tolerance of 6-mercaptopurine (6-MP) therapy in adult acute lymphoblastic leukemia (ALL).

**Methods:**

A total of 216 adult patients who were diagnosed with ALL and treated with cyclophosphamide, cytarabine, and 6-MP [complementary and alternative medicine (CAM) regimen] from September 2015 to December 2019 were included. Polymorphisms were detected by TaqMan SNP Genotyping Assay. Combined with clinical data, the influence of genetic polymorphism on the tolerance of 6-MP in the treatment of ALL was analyzed.

**Results:**

Among the 216 patients, 185 (85.65％) patients had B-ALL and 31 (14.35％) patients had T-ALL. 216 (100％) patients had CC genotype for both TPMT*2 rs1800462 and TPMT*3B rs1800460. The number of TT and TC genotypes for TPMT*3C rs1142345 was 209 (96.76％) and 7 (3.24％), respectively. The allele frequency was 1.62％ for TPMT*3C rs1142345. The number of CC, CT, and TT genotypes for NUDT15 rs116855232 was 166 (76.85％), 48 (22.22％), and 2 (0.93％), respectively. The allele frequency was 12.04％ for NUDT15 rs116855232. The TPMT*3C rs1142345 mutant group (TC+CC genotype) had less transfusion volume of packed red blood cell than the wild group (CC genotype) (*P*＝0.036), and the mutant group (TC+CC genotype) had a higher risk to develop hepatotoxicity (increased aspartate aminotransferase) than the wild group (CC genotype) (*OR*＝9.559, 95％ *CI* 1.135–80.475, *P*＝0.038). The durations of white blood cells (WBC)<1×10^9^/L and absolute neutrophil count (ANC)<0.5×10^9^/L in the NUDT15 rs116855232 mutation group (CT+TT genotype) were longer than that in the wild group (CC genotype) (*P*＝0.005, *P*＝0.007), and the transfusion volume of apheresis-derived platelets in the mutant group (CT+TT type) was greater than that in the wild group (CC genotype) (*P*＝0.014).

**Conclusion:**

Genetic polymorphism of TMPT and NUDT15 has an effect on the tolerance of 6-MP in the treatment of adult ALL. Detecting genotypes of patients with ALL before treatment helps to optimize the dosage of 6-MP, which may help shorten the bone marrow suppression duration and reduce blood transfusion volume.

急性淋巴细胞白血病（ALL）是一种以骨髓和外周血中原始淋巴细胞增生为特征的血液系统恶性肿瘤[1]。硫嘌呤类药物，包括硫唑嘌呤及其类似物6-巯基嘌呤（6-MP），具有抗嘌呤代谢作用，早在1953年就被批准用于ALL的治疗[2]。6-MP是ALL巩固强化及维持治疗中最常用的药物之一，在巩固强化治疗中主要和环磷酰胺、阿糖胞苷组成CAM方案。但是6-MP的治疗指数较窄，其主要不良反应为骨髓抑制和肝损害[2]。既往研究认为硫嘌呤甲基转移酶（TPMT）的遗传变异可导致ALL治疗期间硫嘌呤的活性代谢产物水平升高和血液学不良反应[3]。因此，美国食品药物监督管理局（FDA）建议在6-MP暴露前进行TPMT基因型检测。但亚洲人群中TPMT等位基因突变频率较低，仅为1％～3％，预测效能有限[4]–[9]。2015年Yang等[10]发现核苷二磷酸酶15（NUDT15）的基因多态性与儿童ALL患者6-MP不耐受密切相关，可作为预测耐受情况的有效指标。本研究探讨TPMT、NUDT15基因多态性对成人ALL患者6-MP耐受性的影响，以期了解治疗前检测患者基因型的意义。

## 病例与方法

1. 病例和治疗方案：2015年9月至2019年12月于我院白血病诊疗中心诊断并接受治疗的216例初诊成人ALL患者纳入研究，诊断采用形态学、免疫学、细胞遗传学和分子生物学（MICM）诊断模式，分型采用WHO 2016 关于前体淋巴细胞肿瘤的诊断分型标准。Ph阳性ALL患者64例（29.63％），Ph阴性ALL患者152例（70.37％）。根据ChiCTR-TNC-09000397，所有初诊ALL患者在诱导治疗达到完全缓解（CR）后，给予CAM方案（环磷酰胺+阿糖胞苷+6-MP）为基础的序贯巩固治疗。Ph阴性ALL联合应用门冬酰胺酶，Ph 阳性ALL患者联合应用酪氨酸激酶抑制剂（TKI）。本研究获得中国医学科学院血液病医院伦理委员会批准（伦理批号：HG2021035-EC-1）。所有入组患者或患者的监护人均签署知情同意书。

2. 观察指标及判定标准：回顾性分析216例ALL CR患者第1周期CAM方案治疗期间的临床资料，系统记录患者开始化疗后4周内的血常规、肝肾功能检查结果，采用美国卫生及公共服务部发布的第5版常见不良事件评价标准（CTCAE v.5.0）对患者的不良反应进行评价和分级，并记录不良反应的最高等级。与化疗前相比，临床检测项目，如WBC、中性粒细胞绝对计数（ANC）、丙氨酸转氨酶（ALT）、天冬氨酸转氨酶（AST）、总胆红素（TBIL）、肌酐（Cr），每升高或降低一个或多个等级被认为存在不良反应。查阅病历，详细记录患者的住院时间、WBC<1×10^9^/L持续时间、ANC<0.5×10^9^/L持续时间、悬浮红细胞输注量、单采血小板输注量。

3. 基因型检测：所有ALL患者初诊时留取骨髓标本，分离单个核细胞，利用QIAamp DNA 试剂盒（德国Qiagen 公司产品）提取基因组DNA。DNA样本均保存于−20 °C。我们选择了涉及6-MP代谢的4个SNP位点（TPMT*2 rs1800462、TPMT*3B rs1800460、TPMT*3C rs1142345、NUDT15 rs116855232），并应用TaqMan SNP基因分型试验来检测患者的基因分型。所有SNP位点的Taqman探针和TaqPath™ ProAmp™ Master Mixes均购自美国赛默飞科技公司。在QuantStudio™ 5 Real-Time PCR 仪上进行上述实验，反应体系如下：每孔10 µl，包含5 µl TaqPath™ ProAmp™ Master Mix (1×）、0.5 µl TaqMan 探针（20×）、2 µl DNA（10～20 ng/µl）、2.5 µl ddH_2_O；反应条件：60 °C 30 s，95 °C 5 min；95 °C 15 s，60 °C 60 s 或 90 s 45个循环，60 °C 30 s。使用QuantStudio™设计和分析软件v1.4.1进行等位基因分析。

4. 统计学处理：统计学处理采用SPSS 25.0 软件完成。使用卡方检验评估216例患者的基因型频率的哈迪-温伯格平衡（HWE）。计量资料不符合正态分布，以中位数（范围）表示，组间比较采用秩和检验；计数资料采用卡方检验或Frsher精确概率法。基因多态性对肝肾损害的影响采用逻辑回归分析。在逻辑回归分析中，以基因分型、性别、年龄、民族、免疫分型、Ph染色体、TKI、体表面积、6-MP剂量为协变量。*P*<0.05为差异有统计学意义。

## 结果

1. 入组患者的基本特征：共纳入216例成人ALL患者，其中男129例（59.72％）、女87例（40.28％）；初诊时中位年龄为30（14～66）岁；汉族207例（95.83％）、回族2例（0.93％）、满族5例（2.31％）、蒙古族2例（0.93％）；B-ALL 185例（85.65％）、T-ALL 31例（14.35％）；Ph染色体阳性患者64例（29.63％）、Ph染色体阴性患者152例（70.37％）（表1）。中位6-MP剂量为700（300～1 400）mg，中位住院时间为22（11～41）d，WBC<1×10^9^/L中位持续时间为7（0～22）d，ANC<0.5×10^9^/L中位持续时间为7（0～21）d，悬浮红细胞中位输注量为4.5（0.0～14.5）U，单采血小板中位输注量为2（0～17）治疗量。化疗后44例（20.37％）患者ALT升高，20例（9.26％）患者AST升高，116例（53.70％）患者TBIL升高，另有2例（0.93％）患者出现Cr升高。

**表1:** 216例成人ALL白血病患者的临床特征

临床特征	参数
性别［例（％）］	
男	129（59.72）
女	87（40.28）
初诊年龄［岁，*M*（范围）］	30（14～66）
年龄分组［例（％）］	
<40岁	147（68.06）
≥40岁	69（31.94）
民族［例（％）］	
汉族	207（95.83）
非汉族	9（4.17）
回族	2（0.93）
满族	5（2.31）
蒙古族	2（0.93）
免疫分型［例（％）］	
B-ALL	185（85.65）
T-ALL	31（14.35）
Ph染色体［例（％）］	
阳性	64（29.63）
阴性	152（70.37）
TKI［例（％）］	
伊马替尼	53（82.81）
达沙替尼	11（17.19）
6-MP剂量［mg，*M*（范围）］	700（300～1400）
住院时间［d，*M*（范围）］	22（11～41）
WBC<1×10^9^/L持续时间［d，*M*（范围）］	7（0～22）
ANC<0.5×10^9^/L持续时间［d，*M*（范围）］	7（0～21）
悬浮红细胞输注量［U，*M*（范围）］	4.5（0.0～14.5）
单采血小板输注量［治疗量，*M*（范围）］	2（0～17）
不良反应［例（％）］	
丙氨酸转氨酶升高	44（20.37）
天冬氨酸转氨酶升高	20（9.26）
总胆红素升高	116（53.70）
肌酐升高	2（0.93）

注：ALL：急性淋巴细胞白血病；TKI：酪氨酸激酶抑制剂；6-MP：6-巯基嘌呤；ANC：中性粒细胞绝对计数

2. 基因分型结果：利用TaqMan探针法对所有入组患者进行基因分型检测，结果如下：TPMT*2 rs1800462 C>G：CC型216例（100.00％）；TPMT*3B rs1800460 C>T：CC型216例（100.00％）；TPMT*3C rs1142345 T>C：TT型209例（96.76％）、TC型7例（3.24％），未检测到纯合突变型（CC型），等位基因突变频率为1.62％。NUDT15 rs116855232 C>T：CC型166例（76.85％）、CT型48例（22.22％）、TT型2例（0.93％），等位基因突变频率为12.04％。TPMT*3C rs1142345和NUDT15 rs116855232基因型频率经哈迪-温伯格平衡检验*P*值均>0.05，符合群体连锁遗传规律。结合临床资料，TPMT rs1142345突变型组（TC+ CC型）7例（3.24％），野生型组（TT型）209例（96.76％），两组之间在性别、免疫分型、6-MP剂量差异均无统计学意义（*P*值均>0.05）（表2）。另外，TPMT*3C rs1142345突变型组（TC型）在非汉族人群中的比例高于汉族，但差异无统计学意义（11.11％ 对2.90％，*P*＝0.261）。NUDT15 rs116855232 突变型组（CT+TT型）50例（23.15％），野生型组（CC型）166例（76.85％），两组之间在性别、民族、免疫分型、6-MP剂量差异均无统计学意义（*P*值均>0.05）；Ph阴性组的突变型比例高于Ph阳性组（27.63％对12.50％, *P*＝0.016）。

**表2:** 基因分型在各组分布情况［例（％）］

基因分型	例数	性别		民族		免疫分型		Ph染色体		6-MP [mg *M* ]
		男	女	汉族	非汉族	B-ALL	T-ALL	阳性	阴性	
TPMT*3C rs1142345 T>C										
TC+CC	7	5（3.88）	2（2.30）	6（2.90）	1（11.11）	6（3.24）	1（3.23）	2（3.13）	5（3.29）	700（700~1400）
TT	209	124（96.12）	85（97.70）	201（97.10）	8（88.89）	179（96.76）	30（96.77）	62（96.88）	147（96.71）	700（300~1400）
NUDT15 rs116855232 C>T										
CT+TT	50	31（24.03）	19（21.84）	48（23.19）	2（22.22）	44（23.78）	6（19.35）	8（12.50）	42（27.63）^a^	700（700~1400）
CC	166	98（75.97）	68（78.16）	159（76.81）	7（77.78）	141（76.22）	25（80.65）	56（87.50）	110（72.37）	700（300~1400）

注：ALL：急性淋巴细胞白血病；6-MP：6-巯基嘌呤。^a^与阳性组比较，*P*＝0.016

3. 基因多态性对患者骨髓抑制相关指标的影响：TPMT*3C rs1142345 突变型组悬浮红细胞中位输注量为1.5（0.0～7.5）U，野生型组为5.0（0.0～14.5）U，差异有统计学意义（*P*＝0.036）。但两组之间在住院时间、WBC<1×10^9^/L持续时间、ANC<0.5×10^9^/L持续时间和单采血小板输注量上差异无统计学意义（*P*值均>0.05）（表3）。

**表3:** TPMT、NUDT15基因多态性对骨髓抑制情况的影响

基因分型	例数	住院时间［d，*M*（范围）］	WBC<1×10^9^/L持续时间［d，*M*（范围）］	ANC<0.5×10^9^/L持续时间［d，*M*（范围）］	悬浮红细胞输注量［U，*M*（范围）］	单采血小板输注量［治疗量，*M*（范围）］
TPMT*3C rs1142345 T>C						
TC+CC	7	24（18~29）	7（0~18）	5（0~17）	1.5（0~7.5）	2（0~3）
TT	209	22（11~41）	7（0~22）	7（0~21）	5.0（0~14.5）^a^	2（0~17）
NUDT15 rs116855232 C>T						
CT+TT	50	23（17~41）	9（0~22）	8（0~21）	5.0（0~12.0）	2（0~6）
CC	166	22（11~40）	7（0~21）	6（0~18）^a^	4.5（0~14.5）	2（0~17）

注：ANC：中性粒细胞绝对计数。^a^与突变型组比较，*P*<0.05

NUDT15 rs116855232突变型组WBC<1×10^9^/L的中位持续时间为9（0～22）d，野生型组为7（0～21）d，两组之间差异有统计学意义（*P*＝0.005）；突变型组ANC<0.5×10^9^/L的中位持续时间为8（0～21）d，野生型组为6（0～18）d（*P*＝0.007）；突变型组单采血小板的中位输注量为2（0～6）治疗量，野生型组为2（0～17）治疗量，经秩和检验突变型组单采血小板输注量多于野生型组，且差异有统计学意义（*P*＝0.014）（表3）。另外，NUDT15 rs116855232 突变型组中位住院时间为23（17～41）d，野生型组为22（11～40）d（*P*＝0.077）。

4. 基因多态性对肝肾功能的影响：TPMT*3C rs1142345突变型组出现肝损害（AST升高）的风险高于野生型组，且差异有统计学意义（*OR*＝9.559, 95％*CI* 1.135～80.475, *P*＝0.038）（表4）。NUDT15 rs116855232 突变型组和野生型组发生肝肾损害的风险差异无统计学意义（*P*>0.05）。

**表4:** TPMT、NUDT15基因多态性对肝肾功能的影响

基因分型	ALT升高		AST升高		TBIL升高		Cr升高	
	*OR* (95% *CI*)	*P*值	*OR* (95% *CI*)	*P*值	*OR* (95% *CI*)	*P*值	*OR* (95% *CI*)	*P*值
TPMT*3C rs1142345 T>C								
TC+CC对TT	3.198 (0.478~21.405)	0.231	9.559(1.135~80.475)	0.038	0.582 (0.117~2.889)	0.508	9.714(0.894~105.517)	1.000
NUDT15 rs116855232 C>T								
CT+TT对CC	0.881 (0.365~2.124)	0.778	1.998(0.706~5.659)	0.192	1.372 (0.686~2.745)	0.371	1.093(0.111~10.745)	1.000

注：ALT：丙氨酸转氨酶：AST：天冬氨酸转氨酶；TBIL：总胆红素；Cr：肌酐

## 讨论

6-MP是ALL治疗中常用的化疗药物。在体内经次黄嘌呤鸟嘌呤磷酸核糖转移酶（HGPRT）、肌苷单磷酸脱氢酶（IMPDH）、鸟苷单磷酸合成酶（GMPS）等代谢为有活性的硫鸟嘌呤核苷酸（6-TGN），可掺入DNA和RNA，干扰核苷酸代谢[2],[11]。另外，6-MP也可通过硫嘌呤甲基转移酶（TPMT）代谢为无活性的6-甲基巯嘌呤（6-MMP）和6-甲基硫鸟嘌呤（6-MTG），竞争性减少6-TGN的生成，从而影响6-MP的药效[2]。6-MP及其代谢产物6-TGNs在抗肿瘤的同时也会导致骨髓抑制、肝损害等毒副反应[2]。

诸多证据表明TPMT的活性与 6-TGN的浓度呈负相关，其基因多态性会影响6-MP的毒性及耐受情况[3],[8],[12]。因此，治疗前检测TPMT基因型对个体化调整6-MP剂量具有重要意义。TPMT基因突变相关的骨髓抑制占所有骨髓抑制患者的25％左右[13]。欧美人群中TPMT突变率（约12％）较高，但骨髓抑制率并不高（约 5％）；亚洲人群中TPMT突变并不常见（约2％），但骨髓抑制率却显著增高（约35％）[4]–[9],[14]–[17]。这提示TPMT的基因型检测可能并不适用于亚洲人群，尚需探索影响6-MP代谢、可能改善硫嘌呤类药物毒性预测的其他因素。本研究中，中国成人ALL患者中未检测到TPMT*3B rs1800460突变型和TPMT*2 rs1800462突变型；TPMT*3C rs1142345突变等位基因频率仅为1.62％，这与文献[6]–[7]报道结果相似。TPMT*3C rs1142345突变型组和野生型组患者在住院时间、WBC<1×10^9^/L的持续时间、ANC<0.5×10^9^/L的持续时间和单采血小板输注量上差异均无统计学意义，仅野生型组的悬浮红细胞输注量多于突变型组（TC+CC型）（*P*＝0.036）。另外，TPMT*3C rs1142345突变型组出现肝损害（AST升高）的风险是野生型组的9.559倍。结果表明，中国人群中TPMT突变频率较低，对6-MP导致的骨髓抑制情况预测效能有限，但可用于预测肝损害（AST升高）。

2014年Yang等[18]首次报道了韩国克罗恩病患者NUDT15 rs116855232基因多态性与硫嘌呤诱导的白细胞减少有关。随后，Yang等[10]证实NUDT15 rs116855232的遗传变异与儿童ALL患者6-MP不耐受密切相关，可作为预测6-M耐受情况的有效指标。NUDT15编码一种核苷二磷酸酶，通过去磷酸化作用水解6-TGN以防止掺入DNA，从而通过减少 DNA 损伤和避免细胞凋亡来保护细胞[19]。NUDT15 rs116855232等位基因突变频率在欧美人群中低于2％[10],[20]，而亚洲人群中突变频率较高：中国为11.6％～15.7％[4]–[5],[21]–[22]，日本为9.8％～16.84％[23]–[25]，韩国为7.9％～12.16％[26],[27]，泰国为8.5％～12.75％[28],[29]，印度约为9.5％[30]。目前越来越多的研究认为NUDT15 rs116855232基因多态性与硫嘌呤类药物相关的不良反应、剂量减少有关[4],[21],[23],[26]–[31]。本组中成人ALL患者NUDT15 rs116855232等位基因突变频率为12.04％，与文献[5],[22]中报道的结果接近。突变型组6-MP剂量与野生型组差异无统计学意义，但WBC<1×10^9^/L持续时间、ANC<0.5×10^9^/L持续时间均长于野生型组。另外，突变型组单采血小板的输注量多于野生型组。结果说明NUDT15 rs116855232突变型组CAM为基础的方案骨髓抑制时间更长，也即突变型组对6-MP的耐受性更差。但没有发现NUDT15 rs116855232 突变型组和野生型组发生肝肾损害的风险存在差异。NUDT15 rs116855232可以用来预测6-MP的骨髓毒性。

综上，在亚洲人群中，TPMT基因突变频率较低，不适用于预测6-MP导致的不良反应。近年发现的NUDT15 rs116855232基因多态性与6-MP导致的不良反应密切相关。因此，建议有条件的医疗机构开展NUDT15 rs116855232基因型检测来评估患者6-MP暴露后出现不良反应的风险以保障患者的用药安全及药效。
